# Regulating closure of the neural tube in humans

**DOI:** 10.7554/eLife.108870

**Published:** 2025-09-16

**Authors:** Leon Qarawani, Jacob H Hanna

**Affiliations:** 1 Department of Molecular Genetics, Weizmann Institute of Science Rehovot Israel

**Keywords:** morphogenesis, neural tube closure, CRISPRi, bioengineering, organoids, stem cells, Human

## Abstract

An organoid-based screening platform that allows one-gene-at-a-time knockdown across a whole tissue has been used to identify the genes that regulate closure of the neural tube in humans.

**Related research article** Huang RE, Anand GM, Megale HC, Chen J, Abraham-Igwe C, Ramanathan S. 2025. Arrayed single-gene perturbations identify drivers of human anterior neural tube closure. *eLife* **14**:RP108224. doi: 10.7554/eLife.108224.

In the early embryo, a flat sheet of neural cells must roll up into a neural tube that will later form the brain and spinal cord. Understanding this process, which is known as neurulation, is important because a failure of the neural tube to close properly can cause devastating birth defects such as spina bifida (O’Rahilly and Müller, 2002; Nikolopoulou et al., 2017). However, finding the genes that regulate neural tube closure in humans has been challenging because human early embryos cannot be studied for ethical and technical reasons, and because results from animal experiments often do not align with human biology (Jacobson, 1991; Karfunkel, 1974). Now, in eLife, Roya Huang, Giridhar Anand, Sharad Ramanathan and colleagues at Harvard University report that they have identified some of the genes that control the closure of the anterior neural tube by utilizing an organoid-based model (Huang et al., 2025).

The work involved overcoming technical obstacles in order to: (i) generate an efficient organoid model; (ii) be able to knock down single genes across a whole organoid in a cost-effective manner. The former was achieved using a combination of stem cells and micropatterning arrays to generate a model that resembles certain aspects of four-week-old human embryos (Zeng et al., 2023). In particular, the model was highly reproducible across the different microwells of the array, with the tissue in the organoid thickening, undergoing apposition, and then closing to form a tube-like structure in nearly every replicate. The latter obstacle was overcome by using a technique called "arrayed CRISPR interference" to effectively knock down genes in the organoids generated.

The guide RNAs needed for CRISPR interference are often delivered into cells by lentiviruses, but this approach often produces mosaics (ie, the guide RNAs are delivered to some cells but not others). To study the impact of single-gene knock downs on neurulation, it is important for the lentiviruses to deliver the guide RNAs to as many of the cells in the organoid as possible. Since entry into a cell depends on a receptor on the basolateral surface of the cell, the lentivirus needs to be delivered before cell polarity starts to emerge (Finkelshtein et al., 2013). Huang et al. took a number of steps to ensure near-uniform delivery. In particular, they developed a method to produce small volumes of high-titer lentivirus and deliver them to large numbers of stem cells in parallel via a silicone insert system at an optimal timepoint of the protocol.

With this platform in hand, Huang et al. screened 77 candidate transcription factors that had been identified from gene expression datasets. Three of the genes stood out: knocking down ZIC2 or SOX11 prevented closure of the anterior neural tube, whereas knocking down ZNF521 had the opposite effect, causing premature closure at multiple sites. This suggests that ZNF521 normally acts as a safeguard to prevent the neural tube from closing prematurely (Figure 1).

**Figure 1.**
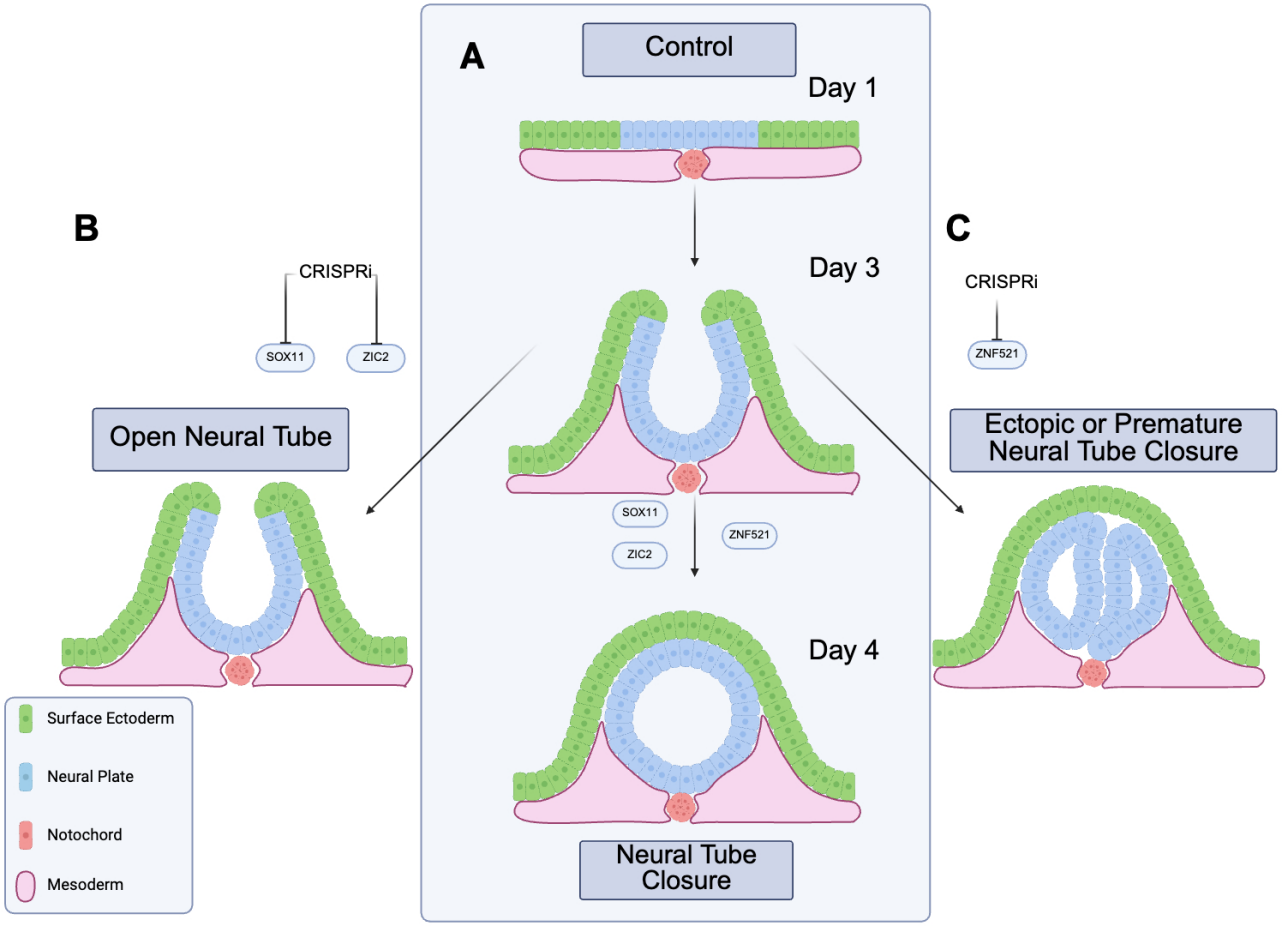
Studying closure of the neural tube in an organoid model. Schematic cross-sections of organoids showing the cells of the neural plate (blue), the surface ectoderm (green), the notochord (red) and adjacent mesodermal tissue (pink). (**A**) In a normal organoid, the neural plate folds and fuses to form a closed neural tube, with a continuous layer of surface ectoderm cells across the top. (**B**) Huang et al. investigated the roles of different genes in this process. When a gene called ZIC2 was knocked down, the neural tube did not close. The same happened when SOX11 was knocked down. (**C**) When a third gene, ZNF521, was knocked down, the neural plate still closed, but it formed multiple discrete closure points (shown here as two separate blue segments), instead of one seamless closure. These phenotypes indicate that ZIC2 and SOX11 promote neural tube closure, whereas ZNF521 prevents aberrant or ectopic closure. Created with BioRender.com.

Further experiments showed that ZIC2 and SOX11 positively co-regulate a set of overlapping neural-plate genes, whereas ZNF521 seems to downregulate many of the genes that are positively regulated by ZIC2 and SOX11. Shared downstream candidate genes included PAX2 and CRABP1, both of which have previously been linked to neural tube defects in animals or humans (Nagai et al., 2000; Elms et al., 2003; Li et al., 2018). Importantly, knocking down just PAX2 or CRABP1 did not prevent closure of the neural tube. This suggests that ZIC2, SOX11 and ZNF521 are at the top of the hierarchy of gene regulation of the neural tube closure process.

This very interesting study delivers two major messages. First, it demonstrates how a uniform approach for one-gene-at-a-time and whole-tissue perturbations in organoid-based models can be made more scalable, which makes it possible to detect meaningful morphogenic outcomes in complex settings. Second, closure of the anterior neural tube in humans seems to be regulated by a coordinated network in which ZIC2 and SOX11 act together to promote adequate closure, while ZNF521 restrains closure at ectopic sites.

The findings of Huang et al. also raise several intriguing questions. ZIC2, SOX11 and ZNF521 appear to be crucial in vitro, but do they also drive neural tube closure in vivo, and can they be implicated in patients with closure defects? And why does SOX11 matter for closure of the anterior neural tube in humans, when mouse mutants do not show similar closure defects (Sock et al., 2004)? Are there human-specific partners, or timing differences across species (Zeng et al., 2023)? Lastly, as organoid-based models grow more sophisticated and robust, researchers could start to integrate biomechanics techniques and/or high-resolution imaging into their experiments to better understand the genetic programs that regulate the folding and closure of the neural tube in humans.
